# Bioorthogonal Non-Canonical Amino Acid Tagging (BONCAT) to detect newly synthesized proteins in cells and their secretome

**DOI:** 10.1371/journal.pone.0329857

**Published:** 2025-08-14

**Authors:** Elizabeth P. Anim, Justin Mezzanotte, Siwei Chu, Ursula Stochaj

**Affiliations:** 1 Department of Physiology, McGill University, Montreal, Quebec, Canada; 2 Quantitative Life Sciences Program, McGill University, Montreal, Quebec, Canada; Shenzhen Bay Laboratory, CHINA

## Abstract

**Background:**

Cells respond to physiological or pathological stimuli by altering the composition of the proteins they produce. This adaptation includes changes to newly translated polypeptides that are destined for intracellular compartments or secretion. The secretome is relevant to cell physiology, as it promotes autocrine, paracrine, and endocrine signaling. These events control cell death, tissue repair, and other regenerative processes. Uncovering the changes in *de novo* protein synthesis under different growth conditions requires reliable methods to identify and quantify newly synthesized proteins. Bioorthogonal Noncanonical Amino Acid Tagging (BONCAT) can generate this information with high spatiotemporal resolution.

**Methods:**

We developed a BONCAT-based protocol to characterize proteins synthesized *de novo* in mammalian cells. Cultured HeLa cells are used as a model system, as their cell physiology is particularly well understood. The current protocol employs L-azidohomoalanine as an L-methionine analog, which is incorporated into newly translated polypeptide chains. After the incubation period, cells and the growth medium, which contains the secretome, are processed separately. Specifically, proteins are alkylated, and L-azidohomoalanine is modified with a biotin affinity tag. Proteins are collected using a rapid precipitation method, which is compatible with the subsequent affinity purification of biotinylated polypeptides. The affinity-purified material can be used for diverse downstream applications, such as Western blotting. Our experiments illustrate the feasibility of different steps of the protocol. Moreover, we discuss potential bottlenecks of the procedure and provide solutions that address these obstacles.

**Discussion:**

Our work demonstrates the power of a modified BONCAT protocol to study newly produced proteins in growing cells and their secretome. This method will be useful to examine the proteome and secretome changes that are linked to the altered performance of cells, tissues, and organs during aging, disease, or other challenging conditions.

## Introduction

Protein homeostasis, also known as proteostasis, requires a balance between protein production and degradation [1,2]. As a critical pillar of the proteostasis network, *de novo* synthesis of polypeptides is essential to align the cellular proteome with changing growth conditions. The proper assessment of protein lifetimes provides fundamental information that is relevant to cell, organ, and organismal health [3]. Appropriate tools and methods to perform these measurements are thus essential to understand mammalian biology and the pathology of a wide variety of diseases.

In eukaryotes, newly synthesized proteins are destined for various different locations. They are targeted to the cytosol, membranes, subcellular organelles and compartments, or secreted into the surroundings of cells. Secreted peptides or proteins shape organismal health through autocrine, paracrine, and endocrine actions [4]. Accordingly, changes in the secretome have been associated with disease or aging [5,6].

Different methods have been developed or improved recently to gain information on the proteome, protein turnover, and newly translated proteins [1,7–10]. Genetic code expansion and incorporation of non-canonical amino acids into peptides and proteins have emerged as effective strategies to advance the field [11]. One of the approaches that are particularly promising relies on the incorporation of non-canonical amino acids into the growing polypeptide chain and subsequent labeling of the non-canonical residue [12–15]. The procedure is suitable for cells growing *in vitro* and a diverse set of model organisms [16]. This approach is employed during Bioorthogonal Non-Canonical Amino Acid Tagging, a method referred to as BONCAT. BONCAT was successfully applied to study diverse proteomes, ranging from mammalian cells and secretomes to soil bacteria [13,15,17–20].

L-Azidohomoalanine (AHA) is a non-canonical amino acid that is taken up by amino acid transporters located in the plasma membrane [21] and incorporated into *de novo* synthesized polypeptides at the position of L-methionine. Using click chemistry, AHA can be covalently linked to a specific tag for the detection and isolation of newly translated proteins [22]. For example, the addition of a biotin moiety to AHA promotes the binding of streptavidin. The subsequent efficient purification of biotinylated proteins relies on the removal of non-incorporated AHA. As well, the proper interpretation of results generated with BONCAT requires a set of control experiments that identify non-specific signals and artifacts. These may include endogenously biotinylated proteins [23,24] or false positives introduced by sample processing.

**Fig 1:**
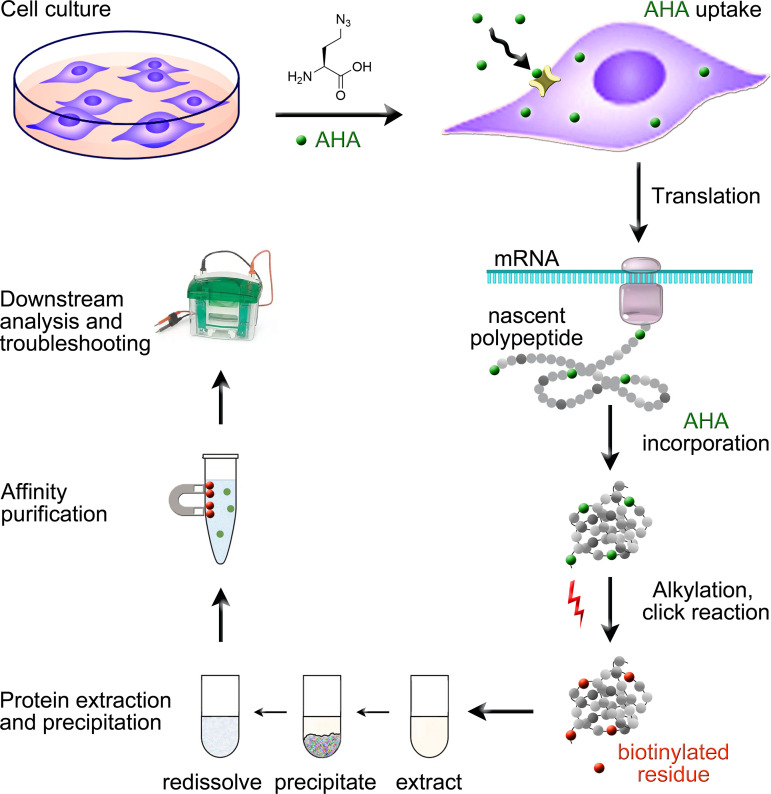
Schematic representation of the workflow for the BONCAT protocol. Cells are cultured in L-methionine-free medium that is supplemented with L-azidohomoalanine (AHA). Transporters residing in the plasma membrane mediate the uptake of AHA into cells. AHA is incorporated into newly synthesized proteins at the position of L-methionine. Cellular fractions and the medium/secretome are collected. Samples are alkylated, and a biotin tag is introduced by click chemistry. Following protein extraction and precipitation, samples are used for affinity purification with magnetic streptavidin beads. Purified proteins are assessed by diverse downstream analyses, such as Western blotting. Control experiments evaluate different steps of the protocol, and troubleshooting is performed, if necessary.

The use of BONCAT to examine *de novo* synthesized intracellular or secreted proteins continues to gain momentum in the basic, applied, and medical fields [25,26]. The protocol developed by us provides a simple and rapid workflow to purify newly translated polypeptides. We address possible bottlenecks of the method and identify potential sources of false positives. Fig 1 depicts a simplified schematic of our protocol.

## Materials and methods

The protocol described in this peer-reviewed article is published on protocols.io, dx.doi.org/10.17504/protocols.io.bp2l6yw5zvqe/v1It and is included for printing as supporting information file 1 (S1 File) with this article.”

Supporting information file 2 (S2 File). Unprocessed Western blots for data shown in Fig 6C.

**Fig 2:**
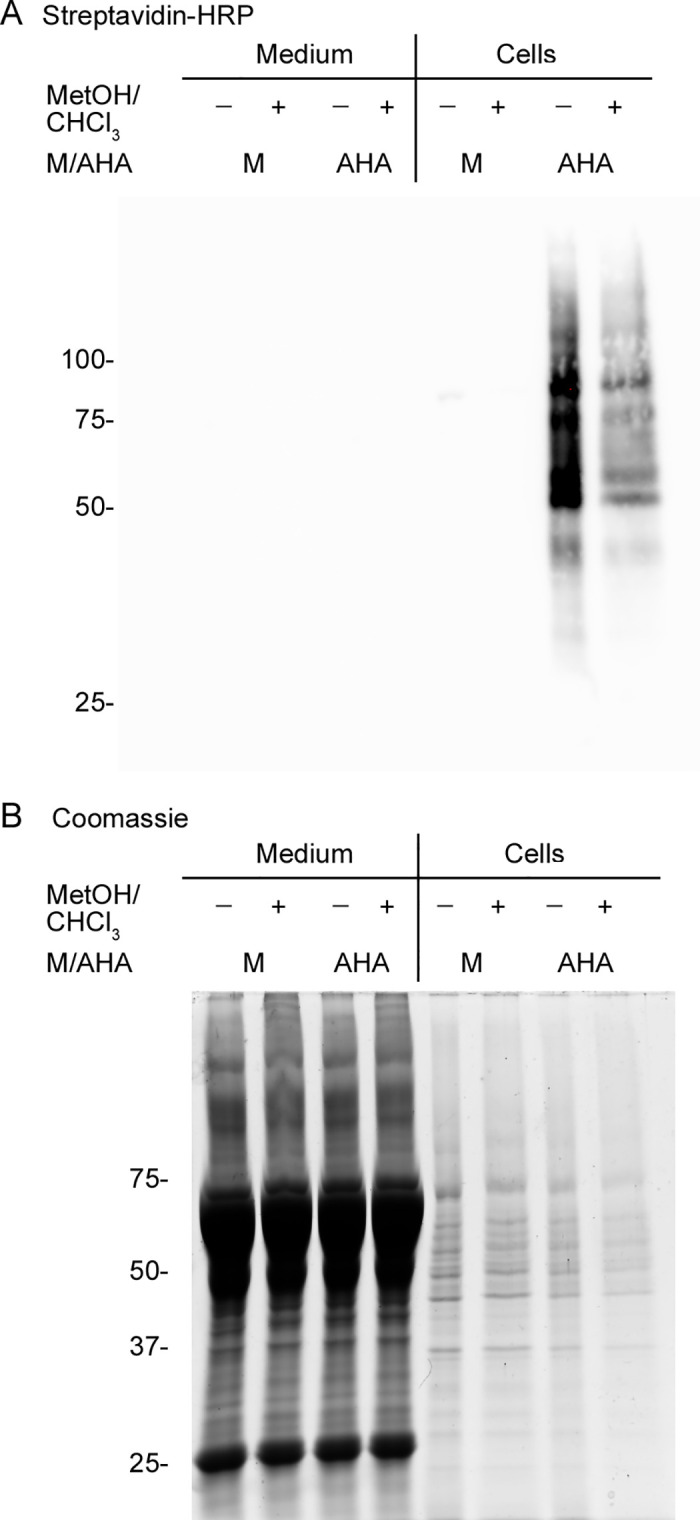
Extraction with methanol/CHCl_3_/water precipitates proteins labeled with the BONCAT method. HeLa cells were incubated with medium containing L-methionine (M) or L-azidohomoalanine (AHA) for 24 **h.** Growth medium and cells were processed as detailed in S1. Medium/secretome and cell fractions were extracted with MetOH/CHCl_3_/water (see details in S1). Aliquots of the samples before and after extraction were separated side-by-side. **(A)** The medium/secretome and cell fractions were separated by SDS-PAGE, blotted onto a nitrocellulose membrane, and probed with horseradish peroxidase (HRP) conjugated to streptavidin (Streptavidin-HRP). Bound Streptavidin-HRP was detected by enhanced chemiluminescence (ECL). **(B)** The same samples shown in part A were separated by SDS-PAGE, and the gel was stained with Coomassie. **(A, B)** The molecular mass of marker proteins (in kDa) is indicated at the left margins.

**Fig 3:**
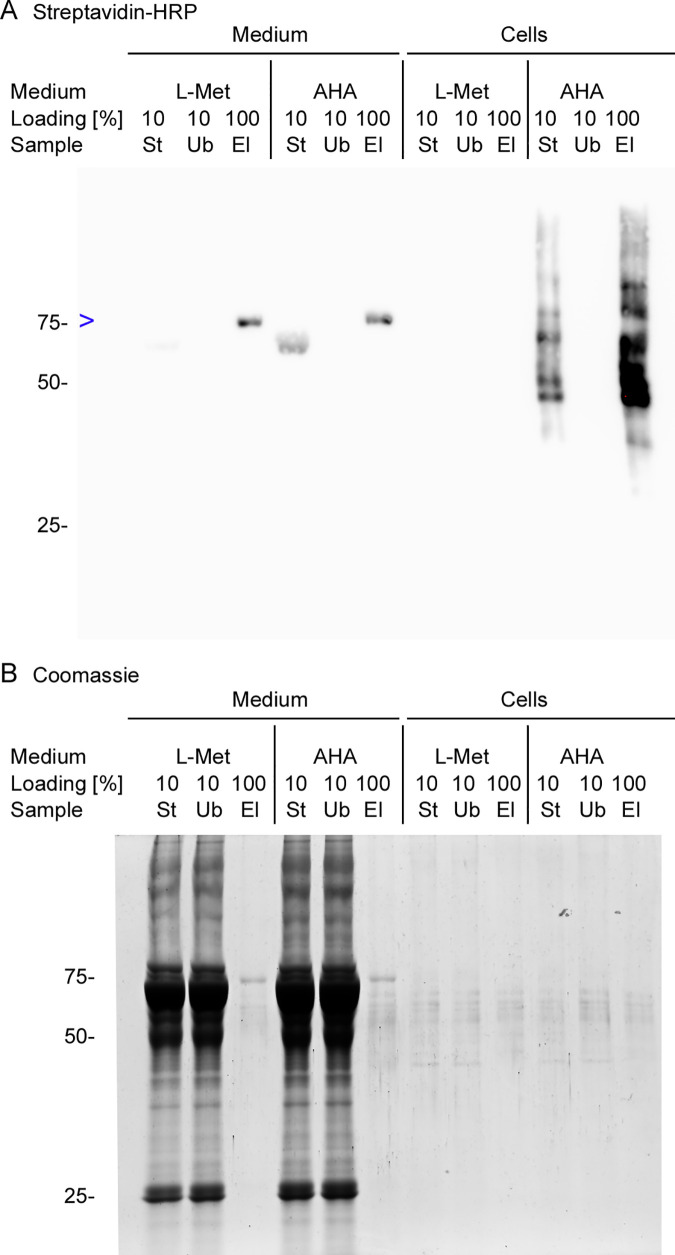
Biotinylated proteins extracted with methanol/CHCl_3_/water are efficiently purified with magnetic streptavidin beads. Cells were cultured in growth medium containing L-methionine (L-Met) or AHA as indicated. **(A, B)** SDS-PAGE, blotting, probing with Strep-HRP, and ECL were performed as described for Fig 2. The position and molecular mass of marker proteins (in kDa) are shown at the left margin. Aliquots of the starting (St), unbound (Ub), and eluted (El) material were analyzed in parallel. **(A)** Streptavidin-binding proteins were identified by blotting. The position of a 75kDa band interacting with Streptavidin-HRP in the medium fraction is marked by a blue arrowhead (>). Note that the band is present both in the medium fraction for cultures that were grown in the presence of L-Met or AHA. **(B)** The samples examined in (A) were separated by SDS-PAGE. Proteins were detected by staining with Coomassie Brilliant Blue (Coomassie).

**Fig 4:**
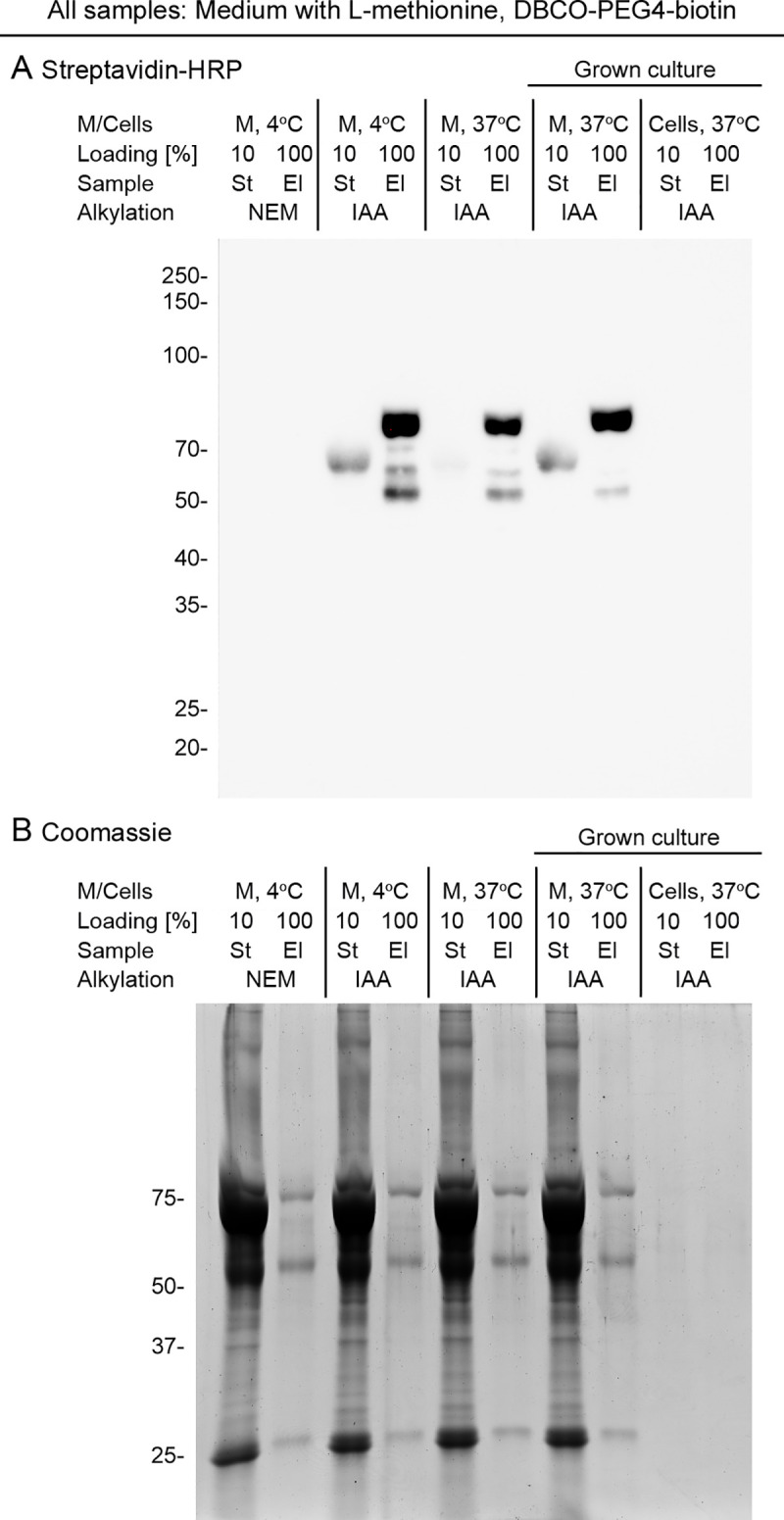
The alkylation agent affects the generation of streptavidin-binding proteins during BONCAT. **(A, B)** For all samples, the medium was supplemented with L-methionine. Culture medium alone was kept at 4ºC or incubated for 24 h at 37ºC. In parallel, the medium/secretome and cell fractions were harvested from cell cultures incubated for 24 h at 37ºC (Grown culture). Material was alkylated with NEM or IAA, as indicated. Following incubation with DBCO-PEG4-biotin, Streptavidin-MagBeads were used for affinity purification. Aliquots of the starting (St), unbound (Ub), and eluted (El) fractions were separated side-by-side. The position and molecular mass of marker proteins in kDa are shown at the left margins. **(A)** Biotinylated proteins were detected with Streptavidin-HRP. **(B)** The same samples analyzed in part (A) were separated by SDS-PAGE, and the gel was stained with Coomassie Brilliant Blue (Coomassie). Note that the ~ 75kDa band pulled down with Streptavidin-MagBeads upon alkylation with IAA was present at low levels or not detectable when NEM was used as alkylating agent.

**Fig 5:**
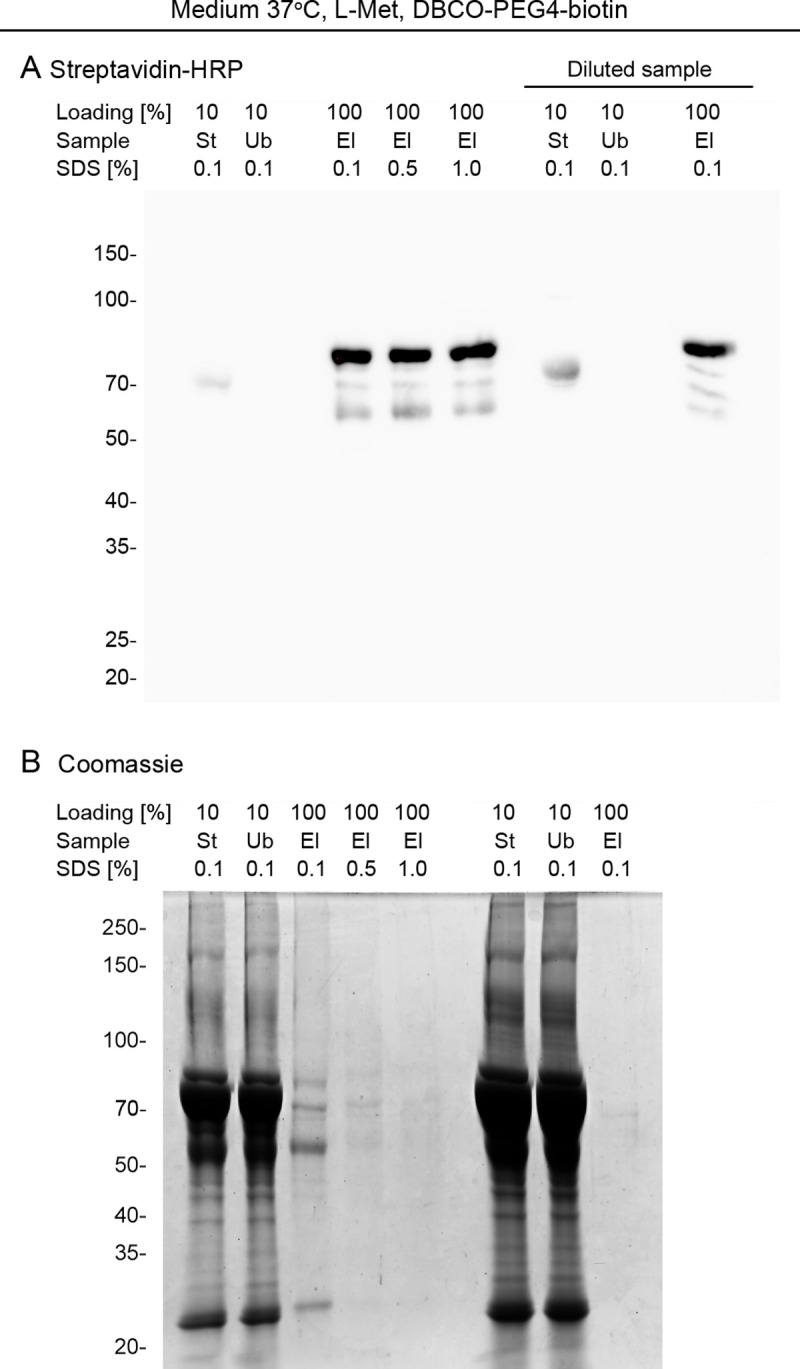
Optimizing wash conditions to reduce non-specific binding to Streptavidin-MagBeads. **(A, B)** Growth medium containing L-methionine was processed with the BONCAT protocol. All samples were treated with IAA and DBCO-PEG4-biotin and then incubated with Streptavidin-MagBeads. Aliquots of the starting (St), unbound (Ub), and eluted (El) material were analyzed side-by-side. The wash buffers contained different concentrations of SDS in PBS, as indicated. The material depicted on the right portion of the gel, labeled Diluted sample, was diluted 1:3 in PBS/0.5% SDS before affinity purification. The position and molecular mass of marker proteins (in kDa) are shown on the left margin. **(A)** Proteins were blotted onto nitrocellulose membranes, and the binding of Streptavidin-HRP was monitored by ECL. **(B)** The same samples analyzed in part A were separated by SDS-PAGE, and proteins were stained with Coomassie Brilliant Blue (Coomassie).

**Fig 6:**
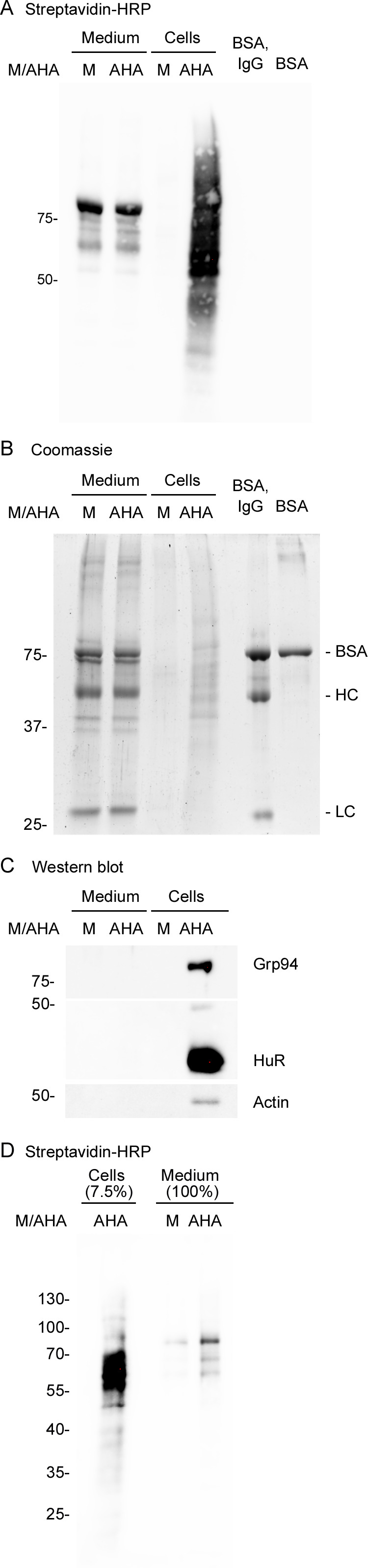
Analysis of proteins purified with Streptavidin-MagBeads. Cells were cultured in medium supplemented with L-methionine (M) or AHA. Medium/secretome and cell fractions were treated with IAA, DBCO-PEG4-biotin, followed by Streptavidin-MagBeads affinity-purification. **(A-C)** Comparable amounts of eluted material were analyzed side-by-side for each part of the figure. The position and molecular mass (in kDa) of marker proteins is indicated at the left margin. **(A)** Samples were blotted onto nitrocellulose filters and probed with Streptavidin-HRP. No signal was detected for bovine serum albumin (BSA) or immunoglobulin G (IgG). **(B)** Samples were separated by SDS-PAGE, and the gel was stained with Coomassie Brilliant Blue (Coomassie). The positions of albumin (BSA), IgG heavy chains (HC) and IgG light chains (LC) are marked at the right side of the gel. **(C)** Material eluted from Streptavidin-MagBeads was examined by Western blotting with different antibodies. Note that Grp94, HuR, and actin are detected in the cell fraction for cultures grown in the presence of AHA. **(D)** Detection of Streptavidin-binding proteins in the medium/secretome fraction. Experiments were carried out as described for part A, but proteins were alkylated with NEM. Biotinylated proteins were detected as in part **A.** The cell fraction contains only 7.5% of the material analyzed for the medium.

### Other materials, solutions, and methods

#### Reagents.

1. Coomassie Brilliant Blue R-250 (Sigma, Catalog #: B-0149)
2. Antibody against Grp94, 9G10 (ENZO, ADI-SPA-850)
3. Antibody against HuR (Santa Cruz Biotechnology, sc-5261)
4. Antibody against actin (Chemicon, Catalog #: Mab 1501)

#### Solutions.

All solutions are prepared in distilled water.

1. Gel staining solution: 0.2% (w/v) Coomassie Brilliant Blue R-250 in 50% (v/v) methanol (MetOH), 10% (v/v) glacial acetic acid. Filter before use.
2. Destainer: 0.7% (v/v) MetOH, 0.5% (v/v) glacial acetic acid.
3. Blocking buffer for Western blots: 5% (w/v) skim milk powder in TBST.
4. Wash buffer for Western blots: TBST.

#### Methods.

1. Processing of PA-gels was carried out with standard protocols. Staining and destaining were performed at room temperature with gentle agitation. In brief, gels were incubated for 30 min in staining solution. After removal of the staining solution, the destainer was added and changed several times until the background was clear. Gels were imaged with a ChemiDoc™ MP Imaging system.
2. Western blotting followed our published procedures [27]. Antibody dilution for Western blotting: Grp94, 1:2,000; HuR, 1:1,000; actin, 1:100,000. ECL signals were collected with a ChemiDoc™ MP Imaging system.
3. Images were processed with Adobe Photoshop CS4.

All images of filters probed with Streptavidin-HRP and Coomassie-stained gels depict the uncropped lanes. Unprocessed filters for Western blotting (Fig 6C) are shown in Supporting information file 2 (S2).

### Expected results

We developed a multistep protocol for the rapid analysis of newly synthesized proteins using BONCAT. The overall workflow is depicted in Fig 1. The sections below show examples of anticipated results and potential pitfalls. We also suggest strategies to overcome obstacles that may arise for different steps of the experiment.

***Removal of free DBCO-PEG4-biotin***. Following the biotinylation of *de novo* synthesized proteins containing AHA and biotin tagging (see Part II, Supp. S1 File), extracts will likely contain non-reacted DBCO-PEG4-biotin. The free agent competes with biotin-modified proteins for the binding to Streptavidin-MagBeads. Therefore, the removal of free DBCO-PEG4-biotin will improve the yield of biotinylated proteins obtained after affinity purification. A combination of methanol (MetOH)/chloroform (CHCl_3_)/water provides a simple and rapid method to precipitate soluble and membrane proteins from detergent-containing solutions [28]. Fig 2 examines whether this technique is appropriate for our BONCAT workflow. To this end, cells were cultured in medium containing L-methionine (M) or AHA, and the medium/secretome and cellular fractions were processed separately (Part II of the protocol, Supp. S1 File). Samples were then assessed before (-) and after (+) extraction with MetOH/CHCl_3_/water. Upon blotting onto nitrocellulose membranes, biotinylated proteins were detected by probing with Streptavidin-HRP (Fig 2A). To visualize the protein profile of individual fractions, the same samples were separated by SDS-PAGE, and the gel was stained with Coomassie (Fig 2B).

Taken together, Fig 2 illustrates that the treatment with MetOH/CHCl_3_/water is suitable to precipitate *de novo* synthesized and other proteins present in the medium/secretome or cell fractions. This is supported by the detection of biotinylated proteins and the overall profile of Coomassie-stained bands.

***Identification of false positives***. The BONCAT method is a sensitive approach to detect and quantify newly synthesized proteins. To limit the misinterpretation of results, it is crucial to identify potential artifacts. We approached this obstacle by comparing material that binds streptavidin for cell cultures that were grown in medium containing L-methionine (L-Met) or AHA. To this end, we conducted Part IIA, IIB, Part III, and Part IV of the BONCAT protocol (Supp. file S1 File). Fig 3 depicts results for the affinity purification. It compares the starting material (St, see Part IV, step 3, Supp. S1 File) with unbound (Ub) and eluted (El) molecules. Note that the loading for starting and unbound material represents 10% of the amount loaded for the eluate. Interestingly, for cells cultured either in the presence of L-methionine or AHA, the medium/secretome fraction contained a prominent ~ 75kDa band that bound to Streptavidin-MagBeads. This resulted in a strong signal when blots were probed with Streptavidin-HRP (Fig 3, blue arrowhead).

In conclusion, protein precipitation with MetOH/CHCl_3_/water is compatible with the downstream affinity purification using Streptavidin-MagBeads. Streptavidin-binding polypeptides are present in the medium/secretome fraction, even for cultures grown in the presence of L-methionine. We further examined the origin of this material with the experiments discussed below.

*The choice of alkylating agent is critical for the processing of BONCAT samples.* As shown in Fig 3, application of the BONCAT protocol revealed false positive material of ~75kDa in the culture medium/secretome of cells grown in the presence of L-methionine. Different scenarios, which are not mutually exclusive, can explain the origin of the biotinylated ~ 75kDa band. Here, we considered the following hypotheses. First, cells biotinylate and secrete the detected polypeptides. (Mammalian cells contain several polypeptides that are biotinylated by endogenous enzymes [23,29]. This includes proteins in the range of 72 - 75kDa [23,29].) Second, the serum used to support cell proliferation contains biotinylated protein(s). Third, enzymatic activities present in the growth medium facilitate protein biotinylation. Fourth, an artifact, introduced during sample processing, causes a modification of the ~ 75kD material that culminates in its binding to streptavidin.

Several lines of evidence argue against the first three scenarios. (1) The ~ 75kD band was identified for L-methionine containing growth medium alone (i.e., in the absence of cultured cells), either kept at 4ºC or incubated at 37ºC for 24 h. This suggests that cellular activities are likely not required to produce the ~ 75kDa streptavidin-binding material. (2) The ~ 75kD streptavidin-binding band was present in L-methionine containing growth medium that was processed for BONCAT (detailed in Part II, Supp S1 File). However, if the processing (Part II, Supp. S1 File) or the incubation with DBCO-PEG4-biotin was omitted, streptavidin-binding ~ 75kDa proteins were not detected (not shown). Collectively, our results suggest that the processing of samples produces ~ 75kDa material in the medium/secretome fraction that strongly interacts with streptavidin. Importantly, we did not observe streptavidin-binding proteins in the cellular fraction of cultures grown in the presence of L-methionine.

Alkylation of amino acid side chains is one of the processing steps of our BONCAT protocol. It is included to prevent the modification of cysteine residues during sample processing. IAA is an alkylation agent that is widely used for the modification of cysteine residues [30]. However, IAA may also react with histidine and lysine side chains [30,31].

While NEM can modify multiple amino acid side chains [31], the agent alkylates cysteine residues with high specificity between pH 6.5 and pH 7.5 [30]. To determine the impact of the alkylating agent, we processed growth medium with NEM and IAA in parallel. All other processing steps were identical (Fig 4). Notably, the ~ 75kDa streptavidin-binding band was present only at low abundance or not observed when the alkylation was performed with NEM. Table 1 depicts the conditions that produced the ~ 75kDa streptavidin-binding band. It should be noted that we did not identify the protein(s) present in the ~ 75kDa band.

**Table 1:** Two parameters are required to observe a strong ~ 75kDa streptavidin-binding band for growth medium supplemented with L-methionine. (Note that AHA was not present for any of the samples.) When combined, the alkylation with IAA and modification with DBCO-PEG4-biotin can generate false positive results for medium alone and the medium/secretome fraction of cultured cells. This is demonstrated by streptavidin binding to material of ~75kDa (Fig 3).

Medium/Cells	IAA	NEM	Without DBCO-PEG4-biotin	With DBCO-PEG4-biotin	Incubation at 37ºC	Incubation at 4ºC
Medium fraction	YES	NO or low signal	NO	YES	NO	NO
Cellular fraction	NO	n. d.	NO	NO	NO	n. d.

IAA, iodoacetic acid; NEM, N-ethylmaleimide; n. d., not determined.

Collectively, results in Fig 3 and 4, summarized in Table 1, emphasize the importance of control experiments that eliminate false positive results. In particular, the choice of the alkylating agent is crucial to limit artifacts related to the growth medium/secretome. By contrast, we did not detect streptavidin-binding proteins in the cell fraction after growth in L-methionine-containing medium and alkylation with IAA (Fig 4, Cells).

*Limiting non-specific binding to Streptavidin-MagBeads.* Several stained bands were present in the material eluted from Streptavidin-MagBeads, when growth medium supplemented with L-methionine was used as the starting material (Fig 4B). These Coomassie-stained non-specific bands of 70-75kDa, ~ 55kDa, and 25kDa, were also present for samples alkylated with NEM. Accordingly, their association with beads may be independent of the interaction with streptavidin. This prompted us to explore conditions that reduce non-specific binding during the affinity-purification. For the control experiments, growth medium containing L-methionine was alkylated with IAA and treated with DBCO-PEG4-biotin. The ~ 75kDa material, which is recognized by Strep-HRP, served as a marker to monitor changes in the enrichment of biotinylated material. Fig 5 shows the conditions used to evaluate non-specific interactions.

Growth medium incubated for 24 h at 37ºC was processed as described in Part IIA and III of the protocol. To reduce non-specific binding, we varied single parameters of the affinity purification (Part IV of the protocol, Supp. S1 File). First, the concentration of SDS in the wash buffer was increased from 0.1% to 0.5%, or 1%. Second, the sample used for affinity purification was diluted 1:3 in PBS/0.5% SDS before addition to Streptavidin-MagBeads; all wash steps were performed with PBS/0.1% SDS. As depicted in Fig 5A, more stringent wash conditions or dilution of the sample prior to affinity purification did not prevent the ~ 75kDa streptavidin-reactive material from binding to the beads. However, increasing the stringency of the wash steps reduced the non-specific binding of 70-75kDa, ~ 55kDa and ~25kDa proteins detected on the Coomassie-stained gel (Fig 5B). Dilution of the sample before affinity-purification also diminished the amount of these proteins in the elution fraction.

Together, these results show that elevating the stringency of the wash buffer or reducing the protein concentration during the binding step decrease non-specific interactions with Streptavidin-MagBeads.

*Combination of the BONCAT protocol with Western blotting to assess the* de novo *synthesis of specific proteins.* BONCAT is often combined with downstream analyses that include mass spectrometry, generating a profile that includes a large number of proteins [9], which is potentially labor-intensive and costly. By contrast, some studies may require BONCAT data for only a limited number of proteins. This information can be generated by Western blot analysis of material eluted from Streptavidin-MagBeads. The experiment shown in Fig 6 illustrates this scenario. Specifically, cells were cultured with growth medium supplemented with L-methionine (M) or AHA, alkylated with IAA, and further processed as detailed in our protocol (Supp. S1 File). Comparable quantities of the eluted material were separated side-by-side in SDS-PA gels and evaluated for the binding of Strep-HRP (Fig 6A), overall protein profile (Fig 6B), and Western blotting with different antibodies (Fig 6C). In particular, the molecular chaperone Grp94, the RNA-binding protein HuR, and actin were examined. We identified all of these candidates as *de novo* synthesized proteins in the cell fraction for cultures grown in the presence of AHA (Fig 6C). Importantly, no ECL signal was obtained when cells were cultured with L-methionine. (It should be noted that due to the use of different antibodies to detect Grp94, HuR, and actin in Fig 6C, the intensities of ECL signals obtained after blotting do not represent the relative abundance of newly synthesized proteins. We expect that the target protein – antibody interactions are specific for each of the antigens tested.)

As described above, several proteins of 70-75kDa, ~ 55kDa, and ~25kDa bound non-specifically to Streptavidin-MagBeads (Fig 4). Given their molecular masses and abundance in serum, we also separated a mixture of bovine serum albumin (BSA) and immunoglobulin G (IgG) or BSA alone on the same gels (Fig 6A, B). No signal was obtained with Streptavidin-HRP (Fig 6A). However, BSA, IgG heavy (HC) and light chains (LC) were clearly visualized with Coomassie staining (Fig 6B). Bands for BSA, IgG HCs and LCs co-migrated with proteins present in the eluate of the medium/secretome fractions. (Note that for Fig 6 larger sample volumes were loaded onto the gels as compared to Figs 2–5. This resulted in stronger signals for the Coomassie-stained gel.). Following alkylation with NEM, probing with Streptavidin-HRP clearly identified bands for the medium/secretome fraction of cells grown in the presence of AHA (Fig 6D). Moreover, we detected HuR in the medium/secretome fraction of AHA-cultured cells, but not for medium supplemented with L-methionine (data not shown).

Collectively, data in Fig 6 demonstrate that our BONCAT workflow is suitable for downstream applications, such as Western blotting. We also present a profile of proteins that non-specifically bind to Streptavidin-MagBeads. While we have not identified these proteins, their electrophoretic mobility is consistent with albumin and IgG, proteins that are especially abundant in serum.

### General notes and troubleshooting

*General notes* Several initial steps are required before the BONCAT protocol is applied.

- Control experiments have to determine how cell viability is affected when L-methionine is substituted for AHA. This informs on the optimal concentration of AHA for the cells or tissues studied.
- AHA incorporation may be low due to residual L-methionine carried over during the transition from methionine-containing medium to AHA-supplemented medium. Such remaining L-methionine may be depleted by keeping cells for a limited amount of time in L-methionine-free medium ( [32], Table 2). However, the use of L-methionine-free medium has to be carefully monitored, as amino acid starvation may trigger a stress response [33,34].
- Proper controls need to (a) identify non-specific modification(s) of proteins, and (b) reduce non-specific binding to Streptavidin-MagBeads. This is especially relevant when medium/secretome samples are examined.

**Table 2:** Potential obstacles for the successful completion of the BONCAT protocols are listed. To optimize the workflow and the quality of results, the proposed solutions can be applied either alone or in combination.

Problem	Solutions
No or low overall incorporation of AHA	(i) Determine toxicity of AHA, adjust concentration, if necessary (ii) Increase the labeling period (iii) Deplete L-methionine before the addition of AHA (iv) Monitor AHA uptake by cells
Poor labeling for protein of interest	(i) Gather information on abundance and turnover of protein of interest in the chosen model system (ii) Add protease inhibitors during sample processing
Binding to streptavidin in the absence of AHA	(i) Determine whether protein is endogenously biotinylated (ii) Use alternative alkylation agent
Non-specific binding of proteins during affinity purification	(i) Increase stringency of wash buffer (ii) Increase number of wash steps (iii) Increase duration of each wash step (iv) Dilute sample before affinity purification (v) Remove albumin and/or IgG before affinity purification (vi) Use alternative method(s) for elution; include free biotin in elution buffer
Low yield after affinity purification	(i) Scale up experiment if conditions tested reduce overall translation (ii) Use sufficient amounts of affinity-beads; monitor unbound material for biotinylated proteins (iii) Optimize incubation time with affinity-beads to ensure efficient binding of biotinylated proteins (iv) Wash steps too stringent; monitor each step for the presence of biotinylated proteins; adjust buffer composition, number and duration of wash steps (v) Use alternative method(s) for elution; include free biotin in elution buffer

*Troubleshooting* We identified potential hurdles that may affect the successful completion of the BONCAT workflow and its downstream applications. Table 2 discusses strategies to address these obstacles.

While not used for the current protocol, kits are commercially available that deplete albumin and/or IgG from complex protein mixtures. For example, albumin is efficiently removed with immobilized dyes or antibodies [35]. Moreover, protein precipitation methods can reduce albumin concentrations in samples containing serum [36], while immobilized protein A/G removes IgG. It should be emphasized that some of the methods used to diminish albumin and/or IgG can also deplete proteins of interest [35].

## Conclusions

This protocol represents a simple and efficient workflow for the purification of *de novo* synthesized proteins that have been labeled with the BONCAT method. We discuss strategies for the processing of cellular and medium/secretome fractions and bottlenecks that have to be considered to optimize the quality of results. We identify potential pitfalls and provide experimental evidence to guide the user towards mitigation. Several modifications to the protocol or additional steps are discussed that can improve data output.

The structured protocol we developed is applicable to a wide variety of experimental conditions. It is suitable for *in vitro* and *in vivo* applications and easy to scale up. Long-term, we expect the protocol to be useful to generate new information on the regulation of proteostasis under different physiological conditions. This includes health and environmental challenges, as well as the response to pharmacological agents and other treatment regimens.

## Supporting information

S1 File Step-by-step protocol, also available on protocols.io. dx.doi.org/10.17504/protocols.io.bp2l6yw5zvqe/v1
(PDF)

S2 File Uncropped images of Western blots. (PDF)

S1 Protocol Bioorthogonal-non-canonical-amino-acid-tagging-bon-gzcqbx2vx. (PDF)

## References

[1] Ross AB, Langer JD, Jovanovic M. Proteome Turnover in the Spotlight: Approaches, Applications, and Perspectives. Mol Cell Proteomics. 2021;20:100016. doi: 10.1074/mcp.R120.002190 33556866 PMC7950106

[2] Alber AB, Suter DM. Dynamics of protein synthesis and degradation through the cell cycle. Cell Cycle. 2019;18(8):784–94. doi: 10.1080/15384101.2019.1598725 30907235 PMC6527273

[3] Fornasiero EF, Savas JN. Determining and interpreting protein lifetimes in mammalian tissues. Trends Biochem Sci. 2023;48(2):106–18. doi: 10.1016/j.tibs.2022.08.011 36163144 PMC9868050

[4] Wu W, Krijgsveld J. Secretome Analysis: Reading Cellular Sign Language to Understand Intercellular Communication. Mol Cell Proteomics. 2024;23(1):100692. doi: 10.1016/j.mcpro.2023.100692 38081362 PMC10793180

[5] Cammarota AL, Falco A, Basile A, Molino C, Chetta M, D’Angelo G, et al. Pancreatic Cancer-Secreted Proteins: Targeting Their Functions in Tumor Microenvironment. Cancers (Basel). 2023;15(19):4825. doi: 10.3390/cancers15194825 37835519 PMC10571538

[6] Hudson HR, Sun X, Orr ME. Senescent brain cell types in Alzheimer’s disease: Pathological mechanisms and therapeutic opportunities. Neurotherapeutics. 2025;22(3):e00519. doi: 10.1016/j.neurot.2024.e00519 39765417 PMC12047392

[7] Shi Y, Weng N, Jian W. Measurement of protein in vivo turnover rate with metabolic labeling using LC-MS. Biomed Chromatogr. 2023;37(7):e5583. doi: 10.1002/bmc.5583 36634055

[8] He F, Aebersold R, Baker MS, Bian X, Bo X, Chan DW, et al. π-HuB: the proteomic navigator of the human body. Nature. 2024;636(8042):322–31. doi: 10.1038/s41586-024-08280-5 39663494 PMC12933382

[9] van Bergen W, Heck AJR, Baggelaar MP. Recent advancements in mass spectrometry-based tools to investigate newly synthesized proteins. Curr Opin Chem Biol. 2022;66:102074. doi: 10.1016/j.cbpa.2021.07.001 34364788 PMC9548413

[10] Tang Q, Chen X. Nascent Proteomics: Chemical Tools for Monitoring Newly Synthesized Proteins. Angew Chem Int Ed Engl. 2023;62(40):e202305866. doi: 10.1002/anie.202305866 37309018

[11] Bhattacharjee R, Lemke EA. Potential vs Challenges of Expanding the Protein Universe With Genetic Code Expansion in Eukaryotic Cells. J Mol Biol. 2024;436(21):168807. doi: 10.1016/j.jmb.2024.168807 39357814

[12] Niu W, Guo J. Cellular Site-Specific Incorporation of Noncanonical Amino Acids in Synthetic Biology. Chem Rev. 2024;124(18):10577–617. doi: 10.1021/acs.chemrev.3c00938 39207844 PMC11470805

[13] Majekodunmi T, Britton D, Montclare JK. Engineered Proteins and Materials Utilizing Residue-Specific Noncanonical Amino Acid Incorporation. Chem Rev. 2024;124(15):9113–35. doi: 10.1021/acs.chemrev.3c00855 39008623 PMC11327963

[14] Jann C, Giofré S, Bhattacharjee R, Lemke EA. Cracking the Code: Reprogramming the Genetic Script in Prokaryotes and Eukaryotes to Harness the Power of Noncanonical Amino Acids. Chem Rev. 2024;124(18):10281–362. doi: 10.1021/acs.chemrev.3c00878 39120726 PMC11441406

[15] Eichelbaum K, Winter M, Berriel Diaz M, Herzig S, Krijgsveld J. Selective enrichment of newly synthesized proteins for quantitative secretome analysis. Nat Biotechnol. 2012;30(10):984–90. doi: 10.1038/nbt.2356 23000932

[16] Kim J-C, Kim Y, Cho S, Park H-S. Noncanonical Amino Acid Incorporation in Animals and Animal Cells. Chem Rev. 2024;124(22):12463–97. doi: 10.1021/acs.chemrev.3c00955 39541258

[17] Bagert JD, Xie YJ, Sweredoski MJ, Qi Y, Hess S, Schuman EM, et al. Quantitative, time-resolved proteomic analysis by combining bioorthogonal noncanonical amino acid tagging and pulsed stable isotope labeling by amino acids in cell culture. Mol Cell Proteomics. 2014;13(5):1352–8. doi: 10.1074/mcp.M113.031914 24563536 PMC4014290

[18] Couradeau E, Sasse J, Goudeau D, Nath N, Hazen TC, Bowen BP, et al. Probing the active fraction of soil microbiomes using BONCAT-FACS. Nat Commun. 2019;10(1):2770. doi: 10.1038/s41467-019-10542-0 31235780 PMC6591230

[19] Shin J, Rhim J, Kwon Y, Choi SY, Shin S, Ha C-W, et al. Comparative analysis of differentially secreted proteins in serum-free and serum-containing media by using BONCAT and pulsed SILAC. Sci Rep. 2019;9(1):3096. doi: 10.1038/s41598-019-39650-z 30816242 PMC6395664

[20] Shin J, Lee C. Profiling of secreted proteins in serum-containing media using BONCAT and pulsed SILAC. In: Luque-Garcia JL. SILAC: Methods and Protocols. New York, NY: Springer US. 2023;235–43. 10.1007/978-1-0716-2863-8_19 36370284

[21] Pelgrom LR, Davis GM, O’Shaughnessy S, Wezenberg EJM, Van Kasteren SI, Finlay DK, et al. QUAS-R: An SLC1A5-mediated glutamine uptake assay with single-cell resolution reveals metabolic heterogeneity with immune populations. Cell Rep. 2023;42(8):112828. doi: 10.1016/j.celrep.2023.112828 37478011

[22] Dieterich DC, Link AJ, Graumann J, Tirrell DA, Schuman EM. Selective identification of newly synthesized proteins in mammalian cells using bioorthogonal noncanonical amino acid tagging (BONCAT). Proc Natl Acad Sci U S A. 2006;103(25):9482–7. doi: 10.1073/pnas.0601637103 16769897 PMC1480433

[23] Grant MKO, Shapiro SL, Ashe KH, Liu P, Zahs KR. A Cautionary Tale: Endogenous Biotinylated Proteins and Exogenously-Introduced Protein A Cause Antibody-Independent Artefacts in Western Blot Studies of Brain-Derived Proteins. Biol Proced Online. 2019;21:6. doi: 10.1186/s12575-019-0095-z 31019379 PMC6474067

[24] Tytgat HLP, Schoofs G, Driesen M, Proost P, Van Damme EJM, Vanderleyden J, et al. Endogenous biotin-binding proteins: an overlooked factor causing false positives in streptavidin-based protein detection. Microb Biotechnol. 2015;8(1):164–8. doi: 10.1111/1751-7915.12150 25211245 PMC4321382

[25] Hatzenpichler R, Scheller S, Tavormina PL, Babin BM, Tirrell DA, Orphan VJ. In situ visualization of newly synthesized proteins in environmental microbes using amino acid tagging and click chemistry. Environ Microbiol. 2014;16(8):2568–90. doi: 10.1111/1462-2920.12436 24571640 PMC4122687

[26] Shah SH, Schiapparelli LM, Yokota S, Ma Y, Xia X, Shankar S, et al. Quantitative BONCAT Allows Identification of Newly Synthesized Proteins after Optic Nerve Injury. J Neurosci. 2022;42(19):4042–52. doi: 10.1523/JNEUROSCI.3100-20.2022 35396330 PMC9097770

[27] Chu S, Joma N, Yong HW, Maysinger D, Kakkar A, Stochaj U. Curcumin and butyrate induce fibroblast senescence without the emergence of fibrosis biomarkers. Aspects of Molecular Medicine. 2023;2:100021. doi: 10.1016/j.amolm.2023.100021

[28] Wessel D, Flügge UI. A method for the quantitative recovery of protein in dilute solution in the presence of detergents and lipids. Anal Biochem. 1984;138(1):141–3. doi: 10.1016/0003-2697(84)90782-6 6731838

[29] Niers JM, Chen JW, Weissleder R, Tannous BA. Enhanced in vivo imaging of metabolically biotinylated cell surface reporters. Anal Chem. 2011;83(3):994–9. doi: 10.1021/ac102758m 21214190 PMC3059349

[30] Hermanson GT. Functional Targets for Bioconjugation. Bioconjugate Techniques. Boston: Academic Press. 2013. 127–228.

[31] Evans CA. Reducing Complexity? Cysteine Reduction and S-Alkylation in Proteomic Workflows: Practical Considerations. Methods Mol Biol. 2019;1977:83–97. doi: 10.1007/978-1-4939-9232-4_7 30980324

[32] Zhang J, Wang J, Ng S, Lin Q, Shen H-M. Development of a novel method for quantification of autophagic protein degradation by AHA labeling. Autophagy. 2014;10(5):901–12. doi: 10.4161/auto.28267 24675368 PMC5119066

[33] Rajanala SH, Ringquist R, Cryns VL. Methionine restriction activates the integrated stress response in triple-negative breast cancer cells by a GCN2- and PERK-independent mechanism. Am J Cancer Res. 2019;9(8):1766–75. 31497357 PMC6726988

[34] Hensen SMM, Heldens L, van Enckevort CMW, van Genesen ST, Pruijn GJM, Lubsen NH. Activation of the antioxidant response in methionine deprived human cells results in an HSF1-independent increase in HSPA1A mRNA levels. Biochimie. 2013;95(6):1245–51. doi: 10.1016/j.biochi.2013.01.017 23395854

[35] Pietrowska M, Wlosowicz A, Gawin M, Widlak P. MS-based proteomic analysis of serum and plasma: Problem of high abundant components and lights and shadows of albumin removal. In: Capelo-Martínez JL. Emerging sample treatments in proteomics. Cham: Springer International Publishing. 2019. 57–76. 10.1007/978-3-030-12298-0_3 31236839

[36] Liu G, Zhao Y, Angeles A, Hamuro LL, Arnold ME, Shen JX. A novel and cost effective method of removing excess albumin from plasma/serum samples and its impacts on LC-MS/MS bioanalysis of therapeutic proteins. Anal Chem. 2014;86(16):8336–43. doi: 10.1021/ac501837t 25083595

