# Supplementary material for: Bioorthogonal Non-Canonical Amino Acid Tagging (BONCAT) to detect newly synthesized proteins in cells and their secretome
Source: PLoS One. 2025 Aug 14;20(8):e0329857. doi: 10.1371/journal.pone.0329857 (PMC12352661; doi:10.1371/journal.pone.0329857)
Supplement: S1 File — dx.doi.org/10.17504/protocols.io.bp2l6yw5zvqe/v1 (PDF) [file pone.0329857.s001.pdf]

May 14, 2025

Bioorthogonal Non-Canonical Amino Acid Tagging (BONCAT) to Detect Newly Synthesized Proteins in Cells and their Secretome

DOI
dx.doi.org/10.17504/protocols.io.bp2l6yw5zvqe/v1

Elizabeth P. Anim[1], Justin Mezzanotte[1], Siwei Chu[1], Ursula Stochaj[1,2]

[1]Department of Physiology, McGill University, Montreal, Quebec, Canada;
[2]Quantitative Life Sciences Program, McGill University

us

Ursula Stochaj
McGill University

OPEN ACCESS

DOI: dx.doi.org/10.17504/protocols.io.bp2l6yw5zvqe/v1

Protocol Citation: Elizabeth P. Anim, Justin Mezzanotte, Siwei Chu, Ursula Stochaj 2025. Bioorthogonal Non-Canonical Amino Acid Tagging (BONCAT) to Detect Newly Synthesized Proteins in Cells and their Secretome. **protocols.io**
https://dx.doi.org/10.17504/protocols.io.bp2l6yw5zvqe/v1

License: This is an open access protocol distributed under the terms of the **Creative Commons Attribution License**, which permits unrestricted use, distribution, and reproduction in any medium, provided the original author and source are credited

Protocol status: Working
We use this protocol and it is working.

Created: April 30, 2025

Last Modified: May 14, 2025

Protocol Integer ID: 218224

Keywords: de novo protein synthesis, non-canonical amino acid, L-azidohomoalanine, biotinylation, affinity purification, secretome analysis, Bioorthogonal Non-Canonical Amino Acid Tagging (BONCAT) , bioorthogonal noncanonical amino acid tagging, changes in de novo protein synthesis, de novo protein synthesis, synthesized protein, produced protein, synthesized proteins in cell, secretome change, secretome cell, protein, de novo in mammalian cell, secretome, composition of the protein, biotinylated polypeptide, translated polypeptide chain, biotin affinity tag, azidohomoalanine, de novo, translated polypeptide, subsequent affinity purification, proteome, secretion, compatible with the subsequent affinity purification, polypeptide chain, methionine analog

Funders Acknowledgements:
NSERC
Grant ID:

Abstract

Cells respond to physiological or pathological stimuli by altering the composition of the proteins they produce. This adaptation includes changes to newly translated polypeptides that are destined for intracellular compartments or secretion. The secretome is relevant to cell physiology, as it promotes a noutocrine, paracrine, and endocrine signaling. These events control cell death, tissue repair and other regenerative processes. Uncovering the changes in *de novo* protein synthesis under different growth conditions requires reliable methods to identify and quantify newly synthesized proteins. Bioorthogonal Noncanonical Amino Acid Tagging (BONCAT) can generate this information with high spatiotemporal resolution.
We developed a BONCAT-based protocol to characterize proteins synthesized *de novo* in mammalian cells. The current protocol employs L-azidohomoalanine as an L-methionine analog, which is incorporated into newly translated polypeptide chains. After the incubation period, cells and the growth medium, which contains the secretome, are processed separately. Specifically, proteins are alkylated, and L-azidohomoalanine is modified with a biotin affinity tag. Proteins are collected using a rapid precipitation method, which is compatible with the subsequent affinity purification of biotinylated polypeptides. The affinity-purified material can be used for diverse downstream applications, such as Western blotting.
Our modified BONCAT protocol was developed to study newly produced proteins in growing cells and their secretome. This method will be useful to examine the proteome and secretome changes that are linked to the altered performance of cells, tissues, and organs during aging, disease, or other challenging conditions.

Guidelines

Follow general laboratory safety practices.

Thumbnail image:

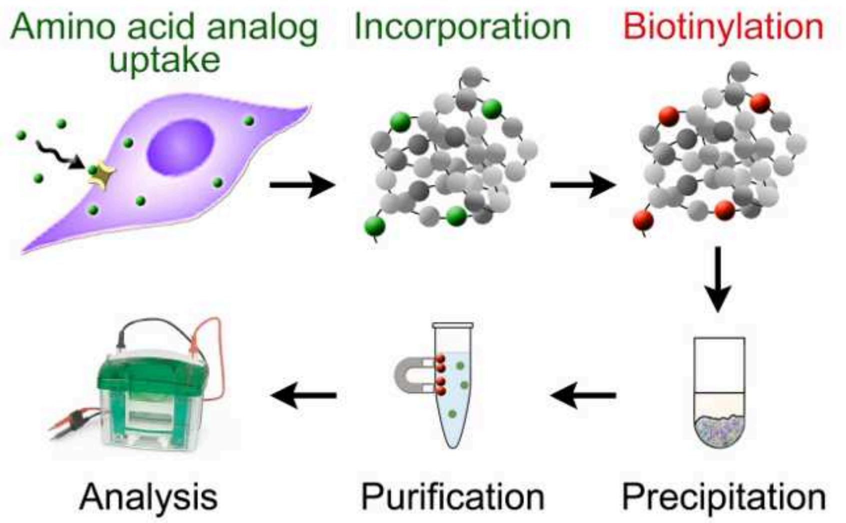

Additional Notes:

1. Store cell extracts and medium/secretome fraction at -70ºC if not used immediately. Avoid freeze-thawing the samples to prevent protein degradation.
2. Prepare AHA stock solution fresh in sterile water.

Materials

Biological materials:

- HeLa cells (ATCC, Catalog #: CCL-2)

Reagents:

1. DMEM, high glucose, no glutamine, no methionine, no cystine **Thermo Fisher Catalog** #21013024
2. 0.05% Trypsin/ 0.53mM EDTA, 1X 500mL **Wisent Bioproducts Catalog** #325-542 CL
3. HEPES 1M Free acid **Wisent Bioproducts Catalog** #330-050-EL
4. 1% Penicillin-Streptomycin **Gibco - Thermo Fisher Scientific Catalog** #15140-122
5. Sodium Pyruvate 100 mM **Wisent Bioproducts Catalog** #600-110-EL
6. L-Glutamine **TCI Chemicals Catalog** #G0063
7. Bovine Calf Serum (BCS) **Fisher Scientific Catalog** #SH3734IR254
8. L-(-)-Cystine Dihydrochloride **TCI Chemicals Catalog** #C0520
9. L-Azidohomoalanine HCl salt **BroadPharm Catalog** #BP-23383
10. DBCO-PEG4-biotin **BroadPharm Catalog** #BP-22295
11. Iodoacetic acid (ICN Biomedicals, Catalog #: 100351)
12. UltraPure™ Sodium Dodecyl Sulfate (SDS) **Thermo Fisher Scientific Catalog** #15525017
13. Methanol, MetOH (ThermoFisher Scientific, Catalog #: BP1105-4)
14. Chloroform, $CHCl_3$, 99.8% (BDH, ACS)
15. Streptavidin MagBeads **Genscript Catalog** #L00936
16. Streptavidin - HRP **Thermo Fisher Scientific Catalog** #434323
17. Nitrocellulose Membrane 0.45 um **Bio-Rad Laboratories Catalog** #1620115
18. SuperSignal™ West Pico PLUS Chemiluminescent Substrate **Thermo Fisher Scientific Catalog** #34580
    or SuperSignal™ West Atto Ultimate Sensitivity Substrate **Thermo Fisher Scientific Catalog** #A38554

Solutions and recipes:

All solutions are prepared in distilled water

- 1M HEPES
- PBS, per L:

A	B
NaCl	8 g
KCl	0.2 g
Na_2HPO_4	1.44 g
KH_2PO_4	0.24 g
Adjust to pH 7.4, autoclave	

- 20% (w/v) SDS in water

- 2% (w/v) SDS in PBS
- L-Azidohomoalanine HCl salt, 50 mM stock solution in sterile water
- Iodoacetic acid (IAA), 200 mM in water; prepare fresh solution for each experiments
- N-ethylmaleimide (NEM), 200 mM in water; prepare fresh solution for each experiments; alternative for IAA
- 1 mM DBCO-PEG4-biotin in water
- Sample buffer for SDS-PAGE, pH 8.0; 2-fold concentrated (2X):

To prepare 10 ml of 2X sample buffer, mix the following

A	B
Tris (hydroxymethyl) amino methane, pH 8.0	1.6 mL
50% glycerol	4.6 mL
SDS	0.4 g
Dithiothreitol	308 mg
0.4% Bromophenol Blue	0.1 mL
β-glycerophosphate	86 mg
Protease inhibitor cocktail, 2X, (Roche, EDTA-free)	
NaF	5mM
NaN_3	2mM
Keep aliquots frozen at -20ºC.	

- Running buffer for SDS-PAGE:

5X stock solution, for 1 L dissolve

A	B
Tris (hydroxymethyl) amino methane	15 g
Glycine	72 g
SDS	5 g
Adjust pH to 8.3 with concentrated HCl	
Keep stock solution at 4 ºC	

- Blotting buffer:

For 700 mL, dissolve in distilled water

A	B
Tris (hydroxymethyl) amino methane	2.13 g
Glycine	10.12 g

- Tris-buffered saline containing 10mM Tris (hydroxymethyl) amino methane, 150 mM NaCl, 0.1 % Tween-20, adjust to pH 7.6 with HCl.

Laboratory equipment:

- BioLite™ Cell Culture Treated Flasks, 75 cm² (ThermoFisher Scientific, Catalog #: 130190)

Equipment	
BioLite™ Cell Culture Treated Flasks, 75 cm2	NAME
Flask	TYPE
Thermo Scientific™	BRAND
130190	SKU
https://www.thermofisher.com/order/catalog/product/130190?SID=srch-srp-130190	LINK

- Tissue culture dish, 35 × 10 mm (Sarstedt, Catalog #: 83.3900)

Equipment	
Tissue culture dish, (ØxH): 35 × 10 mm, surface: Standard	NAME
Culture plates	TYPE
Sarstedt	BRAND
83.3900	SKU
https://www.sarstedt.com/en/products/laboratory/cell-tissue-culture/cultivation/product/83.3900/	LINK

- Hermle, Labnet tabletop centrifuge
- Microfuge (Eppendorf 5424/5424R, Catalog #: EP5404000537)
- Bio-Rad Mini-PROTEAN Cell Gel System
- Bio-Rad Mini Trans-Blot Electrophoretic Transfer Cell
- Sonicator bath; ultrasonic frequency 40 kHz (Codyson, Model PS-10A)
- ChemiDoc™ MP Imaging System (Bio-Rad, Catalog #: 17001401)

Safety warnings

Safety Warnings

- Several of the compounds used in this protocol are hazardous to human health. The list below is not complete. PubChem (**https://pubchem.ncbi.nlm.nih.gov**) provides additional information. Safety Data Sheets should be consulted prior to the application of the protocol.
- Iodoacetic acid is corrosive and acute toxic. N-ethylmaleimide (NEM) is corrosive, acute toxic, and an irritant. Chloroform is a health hazard with acute toxicity. Sodium dodecyl sulfate is flammable, corrosive, and an irritant.

Before start

Determine the possible toxicity of L-azidohomoalanine for the experimental model system to be used. Adjust the final concentration of AHA, if necessary.

Part I. Cell culture conditions for the incorporation of AHA and control experiments

1 **BONCAT medium**

Thaw sterile aliquots of BCS and Pen/Strep.

2 Warm L-glutamine solution in water bath (30°C - 32°C); vortex repeatedly until solution is clear.

3 Supplement 500 mL of DMEM with:

A	B
L-glutamine stock (final concentration: 4 mM)	5 mL
L-cystine (final concentration: 0.201 mM)	31.5 mg
1 M HEPES buffer	5 mL

4 Adjust pH to 7.1, add HCl dropwise.

5 Filter the prepared medium into a sterile bottle using a vacuum-driven filter system.

6 Add BCS (8% final concentration), 5 mL Pen/Strep, and 5 mL of [M] 100 millimolar (mM) sodium pyruvate aseptically.

7 Mix content thoroughly and store at 4 °C until use.

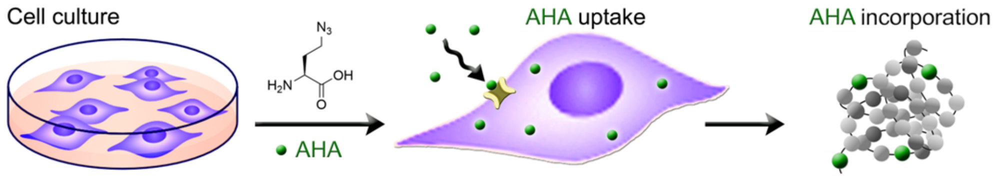

Part I/II: Cells are grown in medium supplemented with AHA. AHA is taken up by cells and incorporated into newly synthesized proteins.

Part II. Biotinylation of AHA-modified polypeptides

3h 5m

8 **A. Treatment of medium/secretome fraction**

8.1 Grow cells in 3 cm dishes, treat cells according to protocol; incubate cells in BONCAT medium containing AHA or supplemented with L-methionine (control samples).

8.2 After AHA incorporation (or L-methionine controls) collect medium (2 mL , contains secretome) into 15 mL tube.

8.3 Spin at 720 x g, Room temperature, 00:05:00 , to remove debris and floating cells. 5m

8.4 Transfer supernatant to fresh 15 mL tube. Do not touch the sediment.

8.5 Add SDS (20% stock solution) to 2% final concentration.

8.6 Add freshly prepared IAA (or NEM) to [M] 50 millimolar (mM) final concentration.

8.7 Rotate 00:30:00 , Room temperature , protect from light. 30m

8.8 Add DBCO-PEG4-biotin to [M] 1 micromolar (µM) final concentration.

Note

The "medium" fraction is approximately 10 times the volume of the "cell" fraction.

8.9 Rotate 01:00:00 , Room temperature , protect from light. 1h

8.10 Store medium at -70 °C or use immediately for MetOH/$CHCl_3$ extraction and affinity-purification with Streptavidin-MagBeads.

9 **B. Treatment of cells**

9.1 After removal of medium, wash dish once with 2 mL PBS, Room temperature .

9.2 Add 100 µL PBS/2% SDS. Scrape material into 1.5 mL tube; Room temperature .

9.3 Rinse dish with 100 µL PBS/2% SDS. Add to test tube; Room temperature .

Note

The solution is highly viscous.

9.4 Add freshly prepared IAA (or NEM) to [M] 50 millimolar (mM) final concentration.

9.5 To shear DNA, sonicate the sample twice for 5 min at RT; protect sample from light. 10m

- To shear DNA, sonicate the sample for 00:05:00 at Room temperature ; protect sample from light. (1/2)
- To shear DNA, sonicate the sample for 00:05:00 at Room temperature ; protect sample from light. (2/2)

9.6 Rotate 00:20:00 at Room temperature , protect from light. **(Total incubation time with IAA is 30 min.)** 20m

9.7 Add DBCO-PEG4-biotin to [M] 10 micromolar (µM) final concentration.

9.8 Rotate 01:00:00 , Room temperature , protect from light. 1h

9.9 Store cell extract at -70 °C or use immediately for MetOH/$CHCl_3$ extraction and affinity-purification with Streptavidin MagBeads.

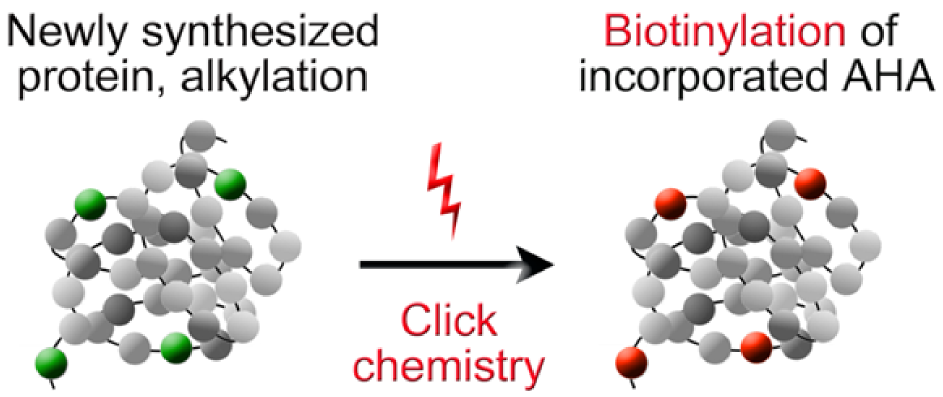

Part II. Click chemistry modifies AHA incorporated into different polypeptides with a biotin moiety.

Part III. Precipitation of proteins present in growth medium/secretome and cell fractions

50m 10s

10

Note

The protocol is described for 100 µL extracts. It can be scaled up for larger volumes. All steps are performed at Room temperature .

Mix 100 µL of extracts described in Part II (Step 8.10 of Part II-A or Step 9.9 of Part II-B) with 400 µL MetOH.

11 Add 100 µL $CHCl_3$, vortex.

12 Add 300 µL distilled water, vortex.

13 Spin 14000 x g, 00:05:00 .

5m

14 Carefully remove the clear upper phase. Do not disturb the white precipitate at interphase.

15 Add 400 µL MetOH, vortex.

16 Spin 20000 x g, 00:05:00 . Carefully remove the clear upper phase. Do not disturb precipitate. 5m

17 Spin 20000 x g, 00:00:10 . Remove residual liquid. 10s

18 Dry sediment at Room temperature for ~ 00:30:00 . 30m

19 Resuspend precipitate in 500 µL PBS/1% SDS.

20 Sonicate two times, 5 min for each step.

20.1 Sonicate, 00:05:00 for each step. (1/2) 5m

20.2 Sonicate, 00:05:00 for each step. (2/2) 5m

21 Proceed to affinity purification or store at -70 °C .

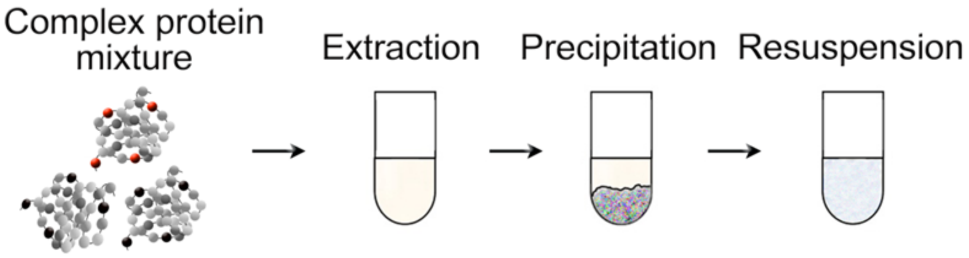

Part III. A complex mixture of proteins includes *de novo* synthesized and biotinylated polypeptides. Proteins are extracted and precipitated. The precipitate is resuspended in an aqueous buffer for subsequent purification.

Part IV. Affinity purification of biotinylated proteins

1h 20m 15s

22 Dilute material precipitated with MetOH/$CHCl_3$ 1:2 with PBS. The sample is now in PBS/0.5% SDS.

23 Spin 15871 x g, Room temperature, 00:05:00 . 5m

24 Use supernatant for incubation with Streptavidin MagBeads.

25 Wash 75 µL of Streptavidin MagBeads three times with 750 µL PBS/0.5% SDS.

26 Add beads to supernatant obtained in Step 24.

27 Incubate 01:00:00 at Room temperature , rotate sample. 1h

28 Spin 00:00:05 in microfuge. 5s

29 Insert into magnet. Aspirate liquid with vacuum.

30 Wash beads three times with 750 µL PBS/0.1% SDS.

31 Add 80 µL 2X sample buffer, vortex, spin 00:00:05 in microfuge, 5s

32 Incubate 00:15:00 at 95 °C . 15m

33 Spin 00:00:05 in microfuge. 5s

34 Insert into magnet and transfer liquid to fresh tube.

35 Separate samples by Sodium Dodecyl Sulfate Polyacrylamide Gel Electrophoresis (SDS-PAGE).

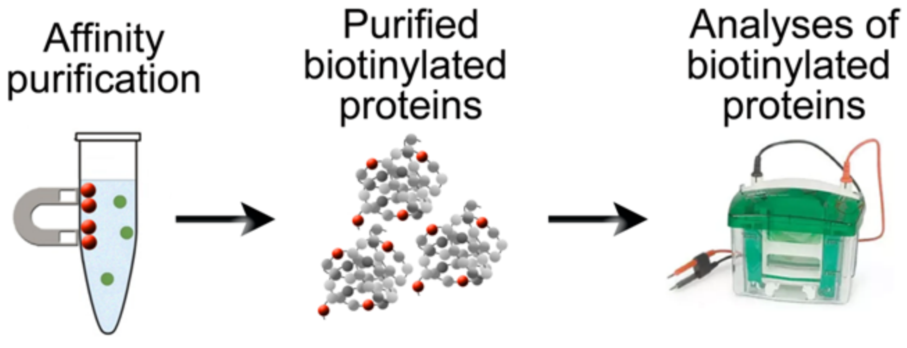

Part IV/V. Affinity purification enriches for biotinylated proteins. These proteins are characterized by different downstream applications.

Part V. Blotting and analysis of de novo synthesized proteins

11h 40m

36 Run samples on SDS-PA gels.

37 Blot proteins onto nitrocellulose filter for 01:20:00 at 76 V, constant voltage. 1h 20m

38 Rinse filter two times for 5 min in TBST.

38.1 Rinse filter for 00:05:00 in TBST. (1/2) 5m

38.2 Rinse filter for 00:05:00 in TBST. (2/2) 5m

39 Block filter in TBST/5% non-fat milk powder, 01:00:00 at Room temperature . 1h

40 Rinse filter two times for 5 min in TBST.

40.1 Rinse filter for 00:05:00 in TBST. (1/2) 5m

40.2 Rinse filter for 00:05:00 in TBST. (2/2) 5m

41 Agitate filter Overnight in cold room with Streptavidin-HRP, diluted 1:5,000 in TBST/1% non-fat milk powder. 8h

42 Wash filter for 01:00:00 in TBST at Room temperature ; change buffer every 15 min. 1h

43 Incubate filter with ECL substrate.

44 Collect ECL signals with ChemiDoc™ MP Imaging system.

Acknowledgements

Funding: Fonds de recherche du Québec, Nature et technologies (FRQNT), Natural Sciences and Engineering Research Council of Canada (NSERC).
