# Supplementary material for: Bioorthogonal Non-Canonical Amino Acid Tagging (BONCAT) to detect newly synthesized proteins in cells and their secretome
Source: PLoS One. 2025 Aug 14;20(8):e0329857. doi: 10.1371/journal.pone.0329857 (PMC12352661; doi:10.1371/journal.pone.0329857)
Supplement: S2 File — (PDF) [file pone.0329857.s002.pdf]

Bioorthogonal Non-Canonical Amino Acid Tagging (BONCAT) to Detect Newly Synthesized Proteins in Cells and their Secretome

Elizabeth P. Anim, Justin Mezzanotte, Siwei Chu, Ursula Stochaj

Uncropped images of Western blots.

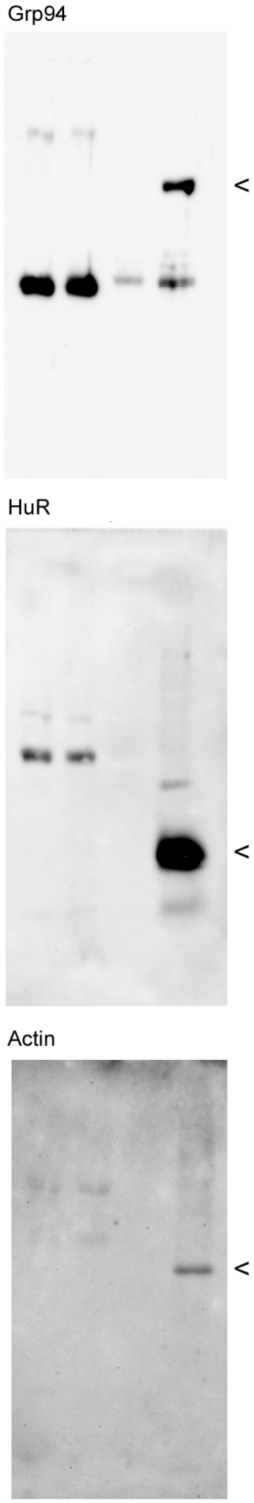

Unprocessed Western blots. Images of the original Western blots are shown for the panels depicted in Fig. 6C. ECL signals are shown after probing the filter with antibodies against Grp94, HuR, and actin. Arrowheads at the right margin indicate the position for each protein of interest.
